# Frederick III., Emperor and King

**Published:** 1888-06-23

**Authors:** 


### FREDERICK III., EMPEROR AND KING.

DIED JUNE 15TH, 1888.

A KING is dead! Yea, truly say a king!
The golden crown we do not always see
On brows so wreathed with heaven-sent sovereignty,
As his whose soul from earth hath taken wing.

His welcome reverently the angels sing
As one whose record of pain nobly borne
Sets e'en above themselves, the sons of morn,
And with his praises all earth's nations ring.

A crown of laurel rested on thy brow,
So brave thou wast in front of battle's blaze;
For thee love's myrtle crown did gladly grow;
Beyond, yet scarce beyond, our tear-dimmed gaze,
Heaven's asphodels shall fitly crown thee now,
Oh, noble monarch of a hundred days!

